# Potentially Pathogenic Free-Living Amoebae Isolated from Soil Samples from Warsaw Parks and Squares

**DOI:** 10.3390/pathogens13100895

**Published:** 2024-10-12

**Authors:** Edyta Beata Hendiger-Rizo, Magdalena Chmielewska-Jeznach, Katarzyna Poreda, Aitor Rizo Liendo, Anna Koryszewska-Bagińska, Gabriela Olędzka, Marcin Padzik

**Affiliations:** Parasitology Laboratory, Department of Medical Biology, Medical University of Warsaw, Litewska 14/16, 00-575 Warsaw, Poland; edyta.hendiger@wum.edu.pl (E.B.H.-R.); magdalena.chmielewska-jeznach@wum.edu.pl (M.C.-J.); poreda.katarzyna@gmail.com (K.P.); arizolie@gmail.com (A.R.L.); anna.koryszewska-baginska@wum.edu.pl (A.K.-B.); marcin.padzik@wum.edu.pl (M.P.)

**Keywords:** Warsaw, soil, free-living amoebae, *Acanthamoeba*, T4 genotype

## Abstract

Free-living amoebae (FLA) are prevalent in diverse environments, representing various genera and species with different pathogenicity. FLA-induced infections, such as the highly fatal amoebic encephalitis, with a mortality rate of 99%, primarily affect immunocompromised individuals while others such as *Acanthamoeba* keratitis (AK) and cutaneous amebiasis may affect immunocompetent individuals. Despite the prevalence of FLA, there is a lack of standardized guidelines for their detection near human habitats. To date, no studies on the isolation and identification of FLA in environmental soil samples in Warsaw have been published. The aim of this study was to determine the presence of amoebae in soil samples collected from Warsaw parks and squares frequented by humans. The isolated protozoa were genotyped. Additionally, their pathogenic potential was determined through thermophilicity tests. A total of 23 soil samples were seeded on non-nutrient agar plates (NNA) at 26 °C and monitored daily for FLA presence. From the total of 23 samples, 18 were positive for FLA growth in NNA and PCR (78.2%). *Acanthamoeba* spp. was the most frequently isolated genus, with a total of 13 positive samples (13/18; 72.2%), and the T4 genotype being the most common. Moreover, *Platyamoeba placida* (3/18; 16.7%), *Stenamoeba berchidia* (1/18; 5.6%) and *Allovahlkampfia* sp. (1/18; 5.6%), also potentially pathogenic amoebae, were isolated. To our knowledge, this is the first report of FLA presence and characterization in the Warsaw area.

## 1. Introduction

Free-living amoebae (FLA), a polyphyletic group of heterotrophic protists, are protozoans with a worldwide distribution [1]. These organisms, known for their ubiquity and opportunistic nature, inhabit various natural environments, including air, freshwater bodies such as ponds, hot springs, lakes, and rivers, as well as saline environments, dust, sediments, and soil [2,3,4,5,6]. Additionally, they have been identified in samples from artificial, anthropogenic settings such as ventilation systems, public swimming pools, bottled mineral water, contact lenses, dialysis units, and dental instruments [7,8]. Moreover, FLA have been documented as part of the natural microbiota of certain fish, mammals, and reptiles [1]. While FLA play a significant role in environmental microbiota, comprehensive research regarding their distribution remains insufficient. Taxonomically, FLA classification remains under investigation, with current classifications relying on morphological, biochemical, and molecular characteristics, thus categorizing pathogenic amoebae into two supergroups: Amoebozoa and Excavata [9].

The life cycle of an amoeba typically comprises two discernible stages: the trophozoite, characterized by active feeding and motility, and the highly resilient cyst stage that can survive in the environment under unfavorable conditions such as lack of food or extreme pH, temperature, and salinity ranges [1,10].

As organisms of varying pathogenicity potential, FLA can pose a threat to human health and life. Diseases caused by FLA are neglected, difficult to diagnose, problematic to treat, and often fatal. Examples of such diseases include *Acanthamoeba* keratitis (AK) and granulomatous amoebic encephalitis (GAE) caused by *Acanthamoeba* spp., primary amoebic meningoencephalitis (PAM) caused by *Naegleria fowleri*, balamuthia amoebic encephalitis (BAE) caused by *Balamuthia mandrillaris* and pulmonary or cutaneous acanthamebiasis [1,11]. Moreover, FLA can act as biological vectors, so-called “Trojan horses”, and can carry various Microsporidia and pathogenic amoeba-resisting bacteria (ARB) such as *Legionella pneumophila*, *Mycobacterium leprae*, and *Pseudomonas* spp. Interactions between FLA and microorganisms exhibit both mutualistic and parasitic dynamics. FLA facilitate the dissemination of microorganisms in the environment, further supporting their growth, as evidenced by studies on *Legionella pneumophila*. Bacteria expressing ARB can successfully survive within both the trophozoite and cyst stages of FLA, possibly posing a threat to human health [10,12,13,14].

Warsaw is a central European metropolis and the capital of Poland belonging to the Mazovia Voivodeship, with a current population of over 1.8 million inhabitants. The city covers an area of 517.2 square kilometers. It is the most densely populated city in Poland (3600 people per square kilometer). Warsaw is located in the umber-transitional climate zone with the influence of both continental and oceanic air masses throughout the year, resulting in highly variable weather patterns in the region [15,16].

Infections caused by FLA may be fatal and life-threatening, especially for immunocompromised people represented by the growing population of individuals with AIDS, organ transplant recipients, cancer, and diabetes patients, as well as pregnant women and children. Despite being considered a rare disease, the global incidence of confirmed FLA infections is steadily increasing. Given all the above, and knowing the challenges in accurate diagnosis and effective treatment of FLA infections, coupled with ongoing climate warming trends, research efforts should be undertaken to assess the risk of FLA infection from the environment [3,17]. Therefore, the aim of this study was to determine the prevalence and potential pathogenicity of FLA in the human environment in the Warsaw area.

## 2. Materials and Methods

### 2.1. Sampling Site

**Figure 1:**
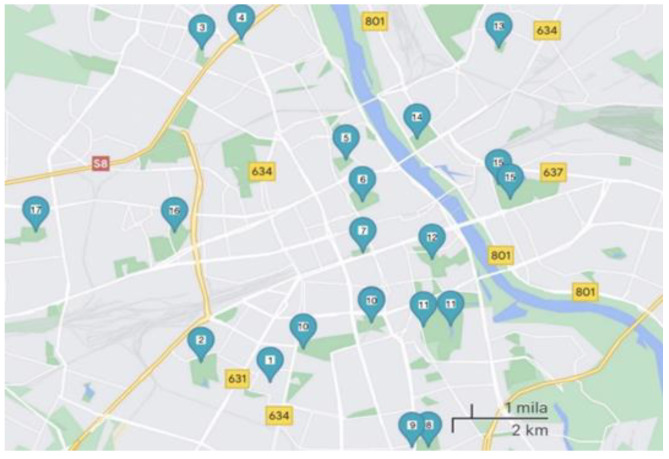
Geographical location of the samples.

All 23 soil samples were taken from parks and squares in the urban agglomeration of Warsaw (Figure 1). The sites for the collection of material for testing were chosen because of the potentially increased risk of contact and, consequently, of FLA infection. These locations are very popular among both residents and tourists. All samples were collected directly from the ground surface using a sterile laboratory spatula into 15 mL sterile falcons in September 2022. During their acquisition, the air temperature oscillated between 9 and 14 °C. The material obtained was stored at 4 °C until further processing in the laboratory.

List of sites from which soil samples were collected:

1. Zaslaw Malicki Park (WAWA)
2. Szczęśliwicki Park (WAWB)
3. Olszyna Park (WAWC)
4. Kaskada Park (WAWD)
5. Krasiński Garden (WAWE)
6. Saski Garden (WAWF)
7. Świętokrzyski Park (WAWG)
8. Arkadia Park (WAWH)
9. Park named after the Home Army ‘Granat’ Group (WAWI)
10. Pola Mokotowskie (2 samples) (WAWJ, WAWK)
11. Royal Łazienki Park (2 samples) (WAWL, WAWM)
12. Marshall Edward Rydz-Smigly Park (WAWN, WAWO)
13. Stefan Wiechecki “Wiecha” Park (WAWP)
14. Praski Park (WAWR)
15. Skaryszewski Park (3 samples) (WAWS, WAWT, WAWU)
16. Edward Szymański Park (WAWW, WAWX)
17. Górczewska Park (WAWY)

### 2.2. FLA Culture

Isolation and cultivation of amoebae was performed at Parasitology Laboratory, Department of Medical Biology, Medical University of Warsaw, Poland. Small amounts of soil samples (about 1 g) were seeded onto an NNA medium at room temperature using a sterile metal spatula. The seeding process was performed close to the flame of the burner, to maintain sterile conditions and to minimize the risk of contamination of the substrate with microorganisms present in the air. Each petri dish was labelled with a letter (corresponding to the sampling location) and the date of the culture, and they were protected by sealing with Parafilm. The plates were incubated at room temperature for 24–48 h, after which the presence of FLA was assessed. If amoebae were suspected, a replicate was performed. The process of creating replicates continued until pure, fungus-free monocultures were obtained.

### 2.3. Thermophilicity

A small piece of agar was applied to the 6-well plate containing the NNA medium with the bottom up from the plates containing the monocultures. All steps were performed close to the flame of the burner. A total of three sets of plates were obtained, each consisting of four six-well plates. The plates were left for two weeks at room temperature, to obtain growth of amoebae throughout the substrate and to obtain the same or similar developmental stage of the isolated FLA. Then, after microscopic verification of the growth of the amoebae, set one was placed at 45 °C, set two at 37 °C, and set three was left at room temperature as a control. The plates were incubated at their respective temperatures for 4 days.

### 2.4. DNA Extraction

Wizard^®^ Genomic DNA Purification Kit was used to extract FLA DNA from amoeba-positive samples. PAS solution was added to a Petri dish and the amoebae were then scraped with a sterile laboratory scraper. The solution was then centrifuged for 10 min at 2500 RPM to obtain a pellet. DNA was isolated from these samples using the instructions provided by the manufacturer under “Isolating Genomic DNA from Tissue Culture Cells and Animal Tissue”.

### 2.5. PCR and Molecular Characterisation of Isolates

PCR amplification of the 18S rRNA gene from the extracted DNA was carried out using two universal primers listed in Table 1. The PCR reactions were performed in 40 cycles with denaturation (95 °C, 30 s), annealing (55 °C, 30 s) and primer extension (72 °C, 1 min) for FLA primers. However, in the case of *Acanthamoeba* spp. primers (JDP-1/2), the PCRs were performed in 40 cycles with denaturation (95 °C, 30 s), annealing (50 °C, 30 s) and primer extension (72 °C, 30 s) [10]. After the last cycles, the primer extension was maintained for 7 min at 72 °C. PCR products were run on a 1% (*w*/*v*) agarose gel in 1xTAE (Tris acetate-EDTA) buffer stained with ethidium bromide and analyzed under UV light using the Gel Doc^TM^ EZ Imager instrument (BIO-RAD). Sequencing of positive PCR products was performed in the Laboratory of DNA Sequencing and Oligonucleotide Synthesis (IBB PAS, Warsaw, Poland). Species identification was based on sequence homology analysis by comparison with the available DNA sequences in the GenBank database.

### 2.6. Phylogenetic Analysis

Phylogenetic analysis based on the partial 18S rRNA gene sequences was performed on the Phylogeny.fr website (https://www.phylogeny.fr/phylogeny.cgi, accessed on 26 February 2024) [20]. After alignment with MUSCLE [21] v3.8.31, ambiguous regions were removed with Gblocks (v0.91b) using the default parameters [22]. The phylogenetic tree was reconstructed using the maximum likelihood method implemented in the PhyML program (v3.1/3.0 aLRT), using the default parameters [23,24]. Graphical representation and editing of the phylogenetic tree were performed with TreeDyn (v198.3.) [25].

## 3. Results

### 3.1. FLA Culture

**Figure 2:**
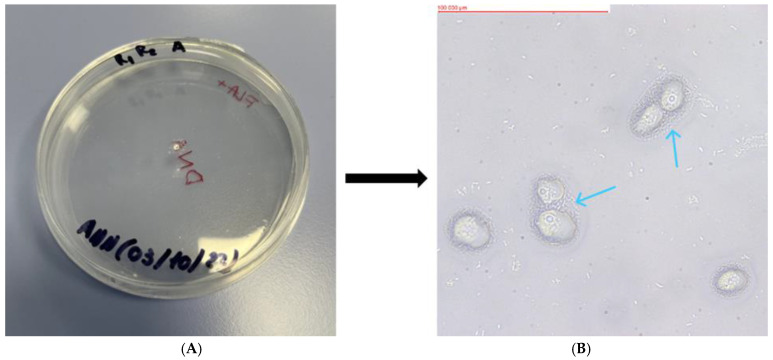
Petri dish containing monoxenic FLA cultures (**A**) and visualized FLA on NNA (**B**) (Sample WAWA: *Platyamoeba placida*). Arrows indicate examples of trophozoites.

**Figure 3:**
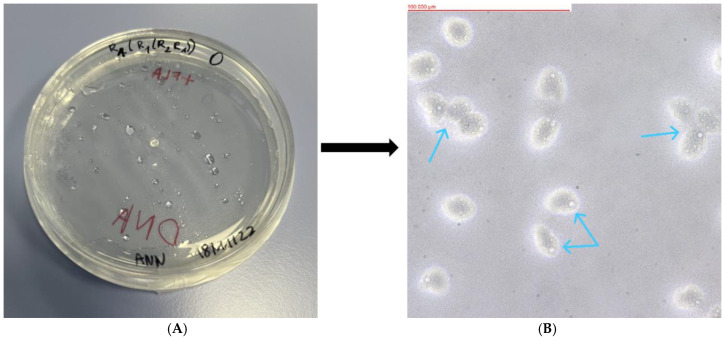
Petri dish containing monoxenic FLA cultures (**A**) and visualized FLA on NNA (**B**) (Sample WAWO: *Acanthamoeba* sp.). Arrows indicate examples of trophozoites.

**Figure 4:**
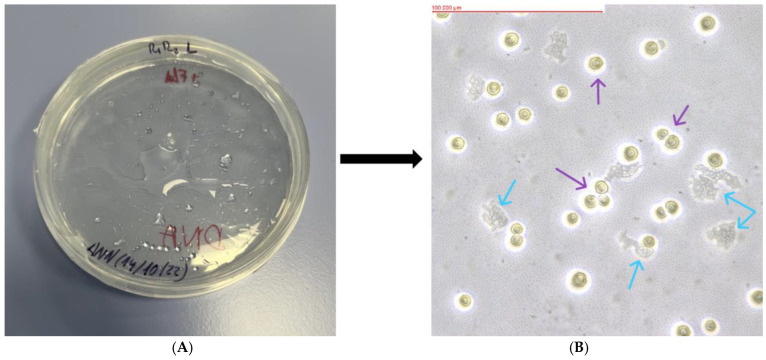
Petri dish containing monoxenic FLA cultures (**A**) and visualized FLA on NNA (**B**) (Sample WAWL: *Allovahlkampfia* sp.). Purple arrows indicate examples of cysts and blue arrows indicate examples of trophozoites.

**Figure 5:**
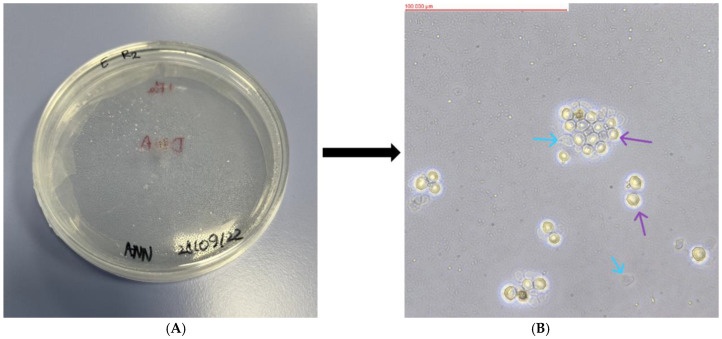
Petri dish containing monoxenic FLA cultures (**A**) and visualized FLA on NNA (**B**) (Sample WAWE: *Stenamoeba berchidia*). Purple arrows indicate examples of cysts and blue arrows indicate examples of trophozoites.

**Figure 6:**
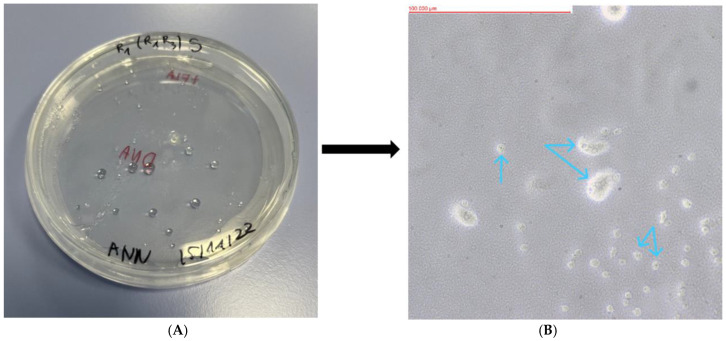
Petri dish containing monoxenic FLA cultures (**A**) and visualized FLA on NNA (**B**) (Sample WAWS: *Acanthamoeba polyphaga*). Arrows indicate examples of trophozoites.

Of the 23 soil samples analyzed, 18 showed the presence of FLA. The mother plates, as well as the replicates formed from them, were assessed using an optical inverted microscope (MW 50 OPTA-TECH, OPTA-TECH, Warsaw, Poland) at 400× magnification. The presence of FLA was visualized using an OPTA-TECH MI5FL 5 MP digital camera (OPTA-TECH, Warsaw, Poland) at 400× magnification (Figure 2, Figure 3, Figure 4, Figure 5 and Figure 6).

### 3.2. Thermophilicity

In plates left at room temperature, no significant morphological changes were observed, the amoebae covered the surface of the substrate more densely, and some trophozoites encysted. The crawlers incubated at 37 °C had mostly transformed into cysts, which were mainly present in groupings of a few/some. Few trophic forms were found, which were characterized by smaller sizes compared to trophozoites in samples residing at room temperature. When incubated at 45 °C, the number of amoebae present as trophozoites was significantly reduced, they were distributed in close proximity to each other and were characterized by smaller sizes compared to trophozoites present in samples incubated at room temperature. Cysts were found in the majority of the samples, forming clusters, and in three of the samples cysts were observed with walls formed into a characteristic star shape. A summary of the results of the experiment is presented in Table 2.

### 3.3. Molecular Characterisation of Isolates

From the total of 23 samples, 18 were positive for FLA growth in NNA and PCR (78.2%) (Table 3). *Acanthamoeba* spp. were the most abundantly isolated species, with a total of 13 samples (13/18; 72.2%), with the T4 genotype being the most common (12/13; 92.3%) followed by *A. polyphaga* (1/13; 7.7%). Additionally, we found *Platyamoeba placida* (3/18; 16.7%), *Stenamoeba berchidia* (1/18; 5.6%) and *Allovahlkampfia* sp. (1/18; 5.6%).

**Figure 7:**
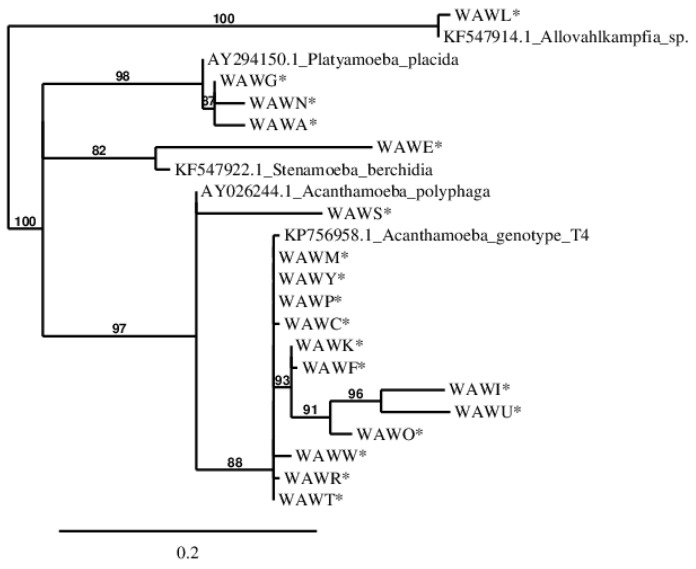
Phylogenetic tree showing the relationship of the 18 newly isolated Amoebozoa strains, based on their partial 18S rRNA gene sequences. Branches with support value smaller than 50% were collapsed. Bootstrap values are shown as % next to the branches. Leaf names with the strains isolated in this study are marked with an asterisk (*). The tree is drawn to scale, with branch lengths measured in the number of substitutions per site (0.2). The GenBank accession numbers of the 18S rRNA sequences used to build the tree are as follow: *Stenamoeba berchidia,* KF547922.1; *Acanthamoeba polyphaga,* AY026244.1; *Allovahlkampfia* sp, KF547914.1; *Platyamoeba placida,* AY294150.1; *Acanthamoeba* genotype T4, KP756958.1.

A total of 18 partial FLA 18S rRNA gene sequences obtained in this study were deposited in the Genbank database. All deposited sequences showed homologies above 89% to DNA sequences present in this database (Table 3). The phylogenetic relationship was inferred by using the maximum likelihood method based on the HKY85 model [23,24]. The gamma shape parameter was estimated directly from the data (gamma = 4.596). Reliability for the internal branch was assessed using the aLRT test (SH-Like). The tree with the highest log likelihood (−1290.58646) is shown in Figure 7.

## 4. Discussion

The above experimental study focuses on the detection and molecular characterization of FLA strains isolated from soil samples. While diseases induced by amoebae are more commonly associated with tropical climates, occurrences, particularly concerning corneal AK infections, have been documented also in temperate regions like Poland, Germany, and Hungary [24,25,26,27,28]. Nevertheless, the absence of specific and effective diagnostic and treatment regimens for FLA-related conditions prompts further research in this area. Environmental research on FLA worldwide includes samples derived mainly from air, water, and soil of both natural and man-made environments [2,4,7,10].

Researchers from Spain have also investigated the occurrence of FLA in soil. The samples analyzed came from beaches, gardens, and agricultural fields on the island of Santiago, part of the Cape Verde archipelago. In total, 26 soil samples were analyzed, with 17 of them containing FLA, representing 65.4% of all samples. Molecular analyses revealed that the predominant genus of amoebae identified was *Acanthamoeba*, representing 82.4% of the FLA detected. Similar results of FLA prevalence and characterization were obtained also in our study [10].

In the densely populated Warsaw agglomeration, no research has been carried out on the occurrence of amoebae in the environment so far. Our findings align with research conducted in 2015 in Poznan, where amoebae belonging to the genus *Acanthamoeba* were isolated from soil samples obtained from sandpits within the city’s playgrounds. However, identification of the genus was conducted only through microscopic examination of trophic forms and cysts observed. The potential pathogenicity was determined by infecting mice, with intranasal inoculation, with a suspension containing isolated amoebae. Among the 13 sandpits examined, pathogenic amoebae were detected in 7, with the FLA exhibiting the highest virulence identified in a sandpit situated near Lake Malta. These findings underscore the importance of continued environmental surveillance and risk assessment to safeguard public health, particularly in recreational areas frequented by children [29].

In a 2014 publication, Adamska et al. presented findings from their study conducted in Poland, focusing on environmental water samples collected from various sources such as rivers, lakes, and marine composting sites. The study revealed the detection of the *Acanthamoeba* genus in 6.5% of the samples and the species *H. vermiformis* in 3.5% of the samples. The study concluded that the most detected genotype of *Acanthamoeba* spp. was T4, which is consistent with the results obtained in this study. Notably, the majority of AK infections in Poland and globally have been associated with this genotype [30]. Similarly, a paper from 2015 authored by Leońska-Duniec et al. investigated the presence of FLA in 86 samples obtained from three distinct water bodies, including outdoor and indoor swimming pools, firefighting reservoirs, fountains, and water networks. The results identified the presence of *Acanthamoeba* genotypes T4, T6, and T16, as well as *H. vermiformis* species. Both studies performed thermophilicity exams that confirmed the potential pathogenicity of 10% and 8.1% of the isolates, respectively [31]. In 2013, Cholewinski et al. published an article focusing on the isolation and assessment of the pathogenicity of amoebae extracted from sand samples obtained from urban sandpits. The study findings revealed the presence of pathogenic amoebae belonging to the *Acanthamoeba* genus in 7 out of 13 samples [32]. 

In our study, the FLA identified predominantly align with the environmental origins of the samples. We isolated *Acanthamoeba* spp., noting that the T4 genotype, commonly linked to human infections, exhibits higher transmissibility and virulence [31,33]. *Acanthamoeba* sp. T4 genotype was the most frequent isolated genus in our samples, with a total of 13 positive samples (72.2%) of which 4 isolates (30.7%) showed increased temperature tolerance. The increased thermotolerance of *Acanthamoeba* spp. correlates with their pathogenicity. Therefore, the ability of *Acanthamoeba* spp. to grow at high temperatures may provide a good indicator of the pathogenic potential of a given isolate. However, the mechanisms by which pathogenic *Acanthamoeba* adapt to high temperatures and sustain metabolic activities require further study [34]. Among other protozoa species identified, *Platyamoeba placida* and *Stenamoeba berchidia* were also sporadically found by other authors [35]. Notably, our research marks the first documentation of *Allovahlkampfia* spp. in soil samples from Poland, a discovery that expands our understanding of FLA distribution. The isolation of *Allovahlkampfia* sp., hitherto known for its elusive presence and challenging treatment profile, underscores its significance. Furthermore, we examined its thermophily profile, which corroborates its potential pathogenicity, presenting a new dimension in studying FLAs and their implications for public health [36,37].

The results obtained from the current study confirm the ubiquitous presence of free-living amoebae in the soil. In our study, 44.5% of isolates showed increased thermophilicity which contrasts with the studies performed by other authors, suggesting potential pathogenicity and raising concerns regarding the development of these diseases.

## 5. Conclusions

The soil analyzed from the Warsaw area serves as a reservoir for FLA, confirming their cosmopolitan nature. *Acanthamoeba* genus was found in most of the isolates with the T4 genotype being the most frequently identified in human infection cases. Moreover, 44.5% of the isolated amoebae cultures exhibited growth at temperatures of 37 °C or above, indicating potential pathogenicity. The presence of pathogenic amoebae in environments close to humans, such as parks and squares, poses a significant threat to human health. However, based on the immunological studies, we know that humans are exposed to free-living amoebae regularly. We still do not fully understand why some people become infected while others do not. There is still room for other free-living amoebae, which have not yet been associated with human diseases, but may be in the future [38]. Therefore, further research should be carried out to determine the epidemiological risk associated with these pathogens and explore strategies for their eradication. Moreover, to our knowledge, this is the first report of potentially pathogenic FLA isolated from soil samples in the Warsaw area.

## Figures and Tables

**Table 1:** Primers used in the study.

Name	5′-3′ Sequence	Tm [°C]	Amplicon Length [bp]	Source
FLA-F	CGCGGTAATTCCAGCTCCAATAGC	55	~800	[18]
FLA-R	CAGGTTAAGGTCTCGTTCGTTAAC			
JDP-1	GGCCCAGATCGTTTACCGTGAA	50	~500	[19]
JDP-2	TCTCACAAGCTGCTAGGGGAGTCA			

**Table 2:** Summary of thermotolerance test results. T—trophozoite, C—cyst, “-”—not detected, “.” impossible to determine the number unequivocally, “ND”—no data (grey color indicates samples in which an increase in the number of trophozoites was found after incubation at a given temperature).

Sample	Room Temperature		37 °C		45 °C	
	Before	After	Before	After	Before	After
WAWA	T = 20	T = 40	T = 10	T = 4	T = 10	T = 30
	-	-	-	C = 10	-	-
WAWC	.	T = 50	T = 10	.	T = 20	.
	-	C = 60	-	-	-	-
WAWE	T = 30	T = 100	T = 30	T = 40	T = 40	T = 6
	-	-	-	C = 40	-	C = 40
WAWF	T = 60	T = 14	T = 20	-	T = 6	T = 5
	C = 80	C = 330	C = 240	C = 240	C = 350	C = 260
WAWG	T = 30	T = 20	T = 20	T = 40	T = 20	T = 30
	C = 40	C = 20	C = 40	C = 40	C = 30	C = 20
WAWI	T = 260	T = 160	T = 70	T = 40	T = 6	T = 140
	C = 30	-	-	C = 10	C = 40	C = 70
WAWK	.	T = 80	.	.	.	.
	-	C = 50	-	.	.	.
WAWL	T = 150	T = 6	T = 10	T = 20	T = 8	T = 30
	C = 170	C = 220	C = 150	C = 210	C = 90	C = 140
WAWM	T = 1	T = 20	-	-	-	-
	C = 40	C = 120	C = 20	C = 70	C = 20	C = 20
WAWN	ND					
WAWO	T = 30	T = 40	T = 20	-	T = 30	-
	C = 9	C = 40	C = 8	C = 100	C = 30	C = 110
WAWP	T = 10	T = 10	T = 5	T = 20	T = 10	-
	C = 80	C = 170	C = 90	C = 150	C = 200	C = 50
WAWR	T = 20	T = 30	T = 15	T = 20	T = 20	-
	C = 90	C = 190	C = 20	C = 230	C = 100	C = 20
WAWS	T = 9	T = 40	T = 10	T = 8	-	-
	C = 1	C = 40	C = 40	C = 20	C = 80	C = 60
WAWT	.	T = 100	T = 50	T = 20	T = 40	-
	-	-	-	-	-	C = 3
WAWU	ND					
WAWW	T = 100	T = 30	T = 20	T = 20	T = 7	-
	C = 120	C = 250	C = 40	C = 120	C = 100	C = 40
WAWY	T = 20	T = 7	T = 40	T = 2	T = 40	-
	C = 120	C = 160	C = 100	C = 130	C = 70	C = 240

**Table 3:** Report of the FLA species isolated from environmental soils of Warsaw parks (NNA: FLA growth in non-nutrient agar culture; PCR: FLA detection by PCR using FLA or JDP primer pair; bp: base pairs; Homology (%) related to NCBI DataBase sequence).

Sample Code	NNA	PCR Primers	Isolated Species	Seq Length (bp)	Identity (%)	ID BLASTn
WAWA	+	FLA	*Platyamoeba placida*	850	>95%	AY294150.1
WAWC	+	JDP	*Acanthamoeb*a sp. T4	426	>98%	KP863877.1
WAWE	+	FLA	*Stenamoeba berchidia*	856	>89%	KF547922.1
WAWF	+	JDP	*Acanthamoeb*a sp. T4	430	>97%	OM414934.1
WAWG	+	FLA	*Platyamoeba placida*	842	>97%	AY294150.1
WAWI	+	JDP	*Acanthamoeb*a sp. T4	416	>89%	MT893156.1
WAWK	+	JDP	*Acanthamoeb*a sp. T4	416	>99%	KP863877.1
WAWL	+	FLA	*Allovahlkampfia* sp.	837	>98%	KF547914.1
WAWM	+	JDP	*Acanthamoeb*a sp. T4	426	>99%	KJ094691.1
WAWN	+	FLA	*Platyamoeba placida*	844	>97%	AY294150.1
WAWO	+	JDP	*Acanthamoeb*a sp. T4	420	>95%	KP756958.1
WAWP	+	JDP	*Acanthamoeb*a sp. T4	424	>98%	KJ094675.1
WAWR	+	JDP	*Acanthamoeb*a sp. T4	435	>98%	LC373013.1
WAWS	+	JDP	*Acanthamoeba polyphaga*	409	>98%	AY026244.1
WAWT	+	JDP	*Acanthamoeb*a sp. T4	426	>99%	KJ094691.1
WAWU	+	JDP	*Acanthamoeb*a sp. T4	434	>87%	OM414922.1
WAWW	+	JDP	*Acanthamoeb*a sp. T4	421	>95%	OP164621.1
WAWY	+	JDP	*Acanthamoeb*a sp. T4	430	>99%	KJ094675.1

## Data Availability

The original contributions presented in the study are included in the article, further inquiries can be directed to the corresponding author.
